# Outcomes of minimal access cytoreductive surgery (M-CRS) and HIPEC/EPIC vs. open cytoreductive surgery (O-CRS) and HIPEC/EPIC in patients with peritoneal surface malignancies: a meta-analysis

**DOI:** 10.1515/pp-2023-0017

**Published:** 2024-03-13

**Authors:** Ajinkya Pawar, Vikas Warikoo, Abhijeet Salunke, Mohit Sharma, Shashank Pandya, Amol Bhardwaj, Sandeep KS, Jebin Aaron

**Affiliations:** Department of Surgical Oncology, 28979 GCRI, Ahmedabad, India

**Keywords:** cytoreductive surgery, HIPEC, EPIC, minimal access surgery, laparoscopic, meta-analysis

## Abstract

**Introduction:**

Minimal Access Surgery (MAS) has shown better peri-operative outcomes with equivalent oncological outcomes in gastrointestinal and thoracic oncology. Open CRS (O-CRS) procedure accompanies inevitable and significant surgical morbidity in patients. The aim of the review article is to compare outcomes of M-CRS and HIPEC/EPIC with open procedure in peritoneal surface malignancies.

**Content:**

Comprehensive search of databases was done and total 2,807 articles were found (2793-PubMed and 14-Cochrane review). PRISMA flow chart was prepared and 14 articles were selected. Meta-analysis was performed according to PRISMA guidelines using random-effects model (DerSimonian Laird) and fixed effect model. Publication bias was tested with Funnel plot and Egger’s regression test. Quality of studies was assessed by Newcastle–Ottawa scale.

**Summary and Outlook:**

Patients in both groups [total (732), M-CRS(319), O-CRS(413)] were similar in demographic characteristics. Peri-operative outcomes were significantly better in M-CRS group in terms of blood loss SMD=−2.379, p<0.001 (95 % CI −2.952 to −1.805), blood transfusion RR=0.598, p=0.011 (95 % CI 0.402 to 0.889), bowel recovery SMD=−0.843, p=0.01 (95 % CI −1.487 to −0.2), hospital stay SMD=−2.348, p<0.001 (95 % CI −3.178 to −1.519) and total morbidity RR=0.538, p<0.001 (95 % CI 0.395 to 0.731). Duration of surgery SMD=−0.0643 (95 % CI −0.993 to 0.865, p=0.892) and CC0 score RR=1.064 (95 % CI 0.992 to 1.140, p=0.083) had no significant difference. Limited studies which evaluated survival showed similar outcomes. This meta-analysis shows that M-CRS and HIPEC/EPIC is feasible and has better peri-operative outcomes compared to open procedure in patients with limited peritoneal carcinoma index (PCI) peritoneal surface malignancies. Survival outcomes were not calculated. Further studies are warranted in this regard.

## Introduction

In the last two to three decades the management of patients with peritoneal surface malignancies has transitioned from palliative care to therapeutic and often curative intent [1]. Cytoreductive surgery (CRS) for removal of macroscopic disease and intra-peritoneal chemotherapy to treat the microscopic remnant disease has become a standard for treatment of peritoneal surface malignancies [2], [3], [4], [5]. Although survival outcomes have improved, these complex surgical procedures accompany inevitable high morbidity and mortality in patients, which is comparable to other high risk open oncological procedures [6, 7]. The morbidity of these procedures can be reduced to an extent by better peri-operative optimisation of patients, still the complication rates are high and management becomes a challenge [8, 9].

The role of minimal access surgery like laparoscopic surgery, hand assisted laparoscopic surgery and robotic surgery has already been established in gastrointestinal and thoracic oncology. The oncological and survival outcomes are similar but the complication rates and morbidity is significantly lower in minimal access surgery [10], [11], [12], [13], [14], [15]. The role of minimal access surgery in peritoneal surface malignancies is evolving rapidly due to early referral of patients to tertiary centres with early stages and low peritoneal burden. Feasibility of laparoscopic CRS and HIPEC in low peritoneal carcinoma index (PCI) patients with low grade and borderline tumours have been described since 2011 [16, 17]. For high grade tumours like ovary and colorectal, literature is limited yet better peri-operative outcomes with minimal access surgery have been described [18].

The purpose of this meta-analysis is to assess the current literature and studies comparing open CRS and intraperitoneal chemotherapy procedure with minimal access CRS and to determine whether minimal access surgery is feasible and has better peri-operative outcomes compared to open procedure.

## Materials and methods

### Search methodology and selection criteria

A comprehensive search of the published literature was done using databases of PubMed and Cochrane review. Key words used for identifying studies for this meta-analysis were ‘Cytoreductive surgery’, ‘HIPEC’, ‘EPIC’, ‘Laparoscopic’, ‘Robotic’ and ‘Minimal access’. Key words and search terms were kept broad so as to encompass all possibilities of studies applicable. Manual search of relevant publications was done to supplement the data. There were no restrictions of the date of publication in the included studies. This search was done on 22/03/2023.

### Data assessment and inclusion and exclusion criteria

After elimination of duplicate abstracts, two investigators independently reviewed all abstracts and full text of articles which were regarded as potentially eligible for further consideration. Hand-searched reference lists of relevant articles were performed to identify further articles for analysis. Thereafter, eligible articles were selected for final analysis according to predefined inclusion and exclusion criteria. Disagreements were resolved by consensus.

**Figure 1:**
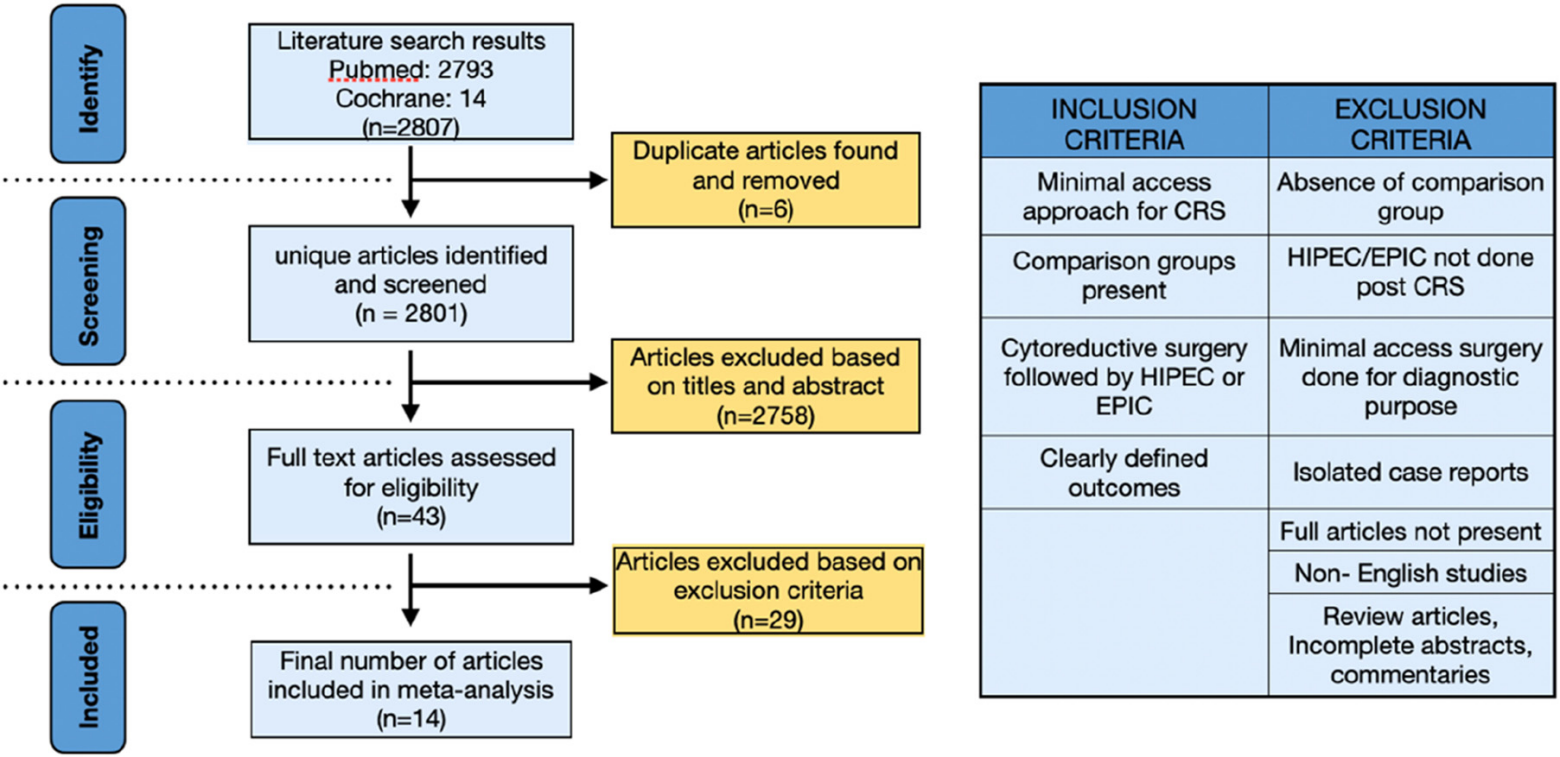
The Preferred Reporting Items for Systematic Review and Meta-Analysis (PRISMA) flowchart.

Studies comparing outcomes of minimal access cytoreductive surgery like laparoscopic cytoreductive surgery, hand-assisted laparoscopic cytoreductive surgery or robotic cytoreductive surgery and HIPEC with open cytoreductive surgery and HIPEC were included. Studies which used EPIC instead of HIPEC were also included. Studies which did not have a comparative or control group with open CRS and HIPEC were not included. Studies with feasibility and peri-operative outcomes were focussed instead of survival outcomes due to limited follow up and data. Further inclusion and exclusion criteria is presented in the table in Figure 1. Authors of studies were not contacted for lack of data or missing data.

### Extraction of data

Data from all finalised articles and studies was extracted and tabulated in google spread sheet under headings as follows: first author, title of study, country, year of publication, study duration, type of minimal access surgery, HIPEC/EPIC, HIPEC/EPIC protocols, demographic characteristics, type of primary, median PCI, CC score, duration of surgery, blood loss, blood transfusion, bowel recovery, post-operative grade 1–4 complications, hospital stay, follow up, mortality, disease free survival and overall survival.

### Assessment of risk of bias

The risk of bias and quality of studies was assessed using Newcastle–Ottawa scale [19]. Two authors independently performed the scores and the results were compared. Agreement was reached by consensus. According to the scale, articles having stars more than five are considered good articles.

### Statistical analysis and data synthesis

The meta-analysis was performed in line with recommendations from the PRISMA (preferred reporting of items for systematic review and meta-analysis) guidelines [20]. A fixed-effect (weighted with inverse variance) or a random-effects model was used where appropriate in our meta-analysis. We evaluated heterogeneity between articles using the χ^2^ and I^2^ statistic with Higgins and Thompsons method. Heterogeneity between articles was assessed to be greater with higher χ^2^ and I^2^. When the p-value was <0.05, the assumption of homogeneity was rejected and a random-effects model of DerSimonian and Laird was adopted. Otherwise, a fixed-effects model of Mantel and Haenszel was used. For calculating publication bias, Funnel plot and Egger’s regression test was used. Comparison of data between M-CRS and control group i.e. O-CRS, was done using relative risk (RR) and standardized mean difference (SMD) as the summary statistic. The point estimate of the RR and SMD was considered to be statistically significant at the p <0.05 level if the 95 % confidence interval did not cross the value of 1 for RR and 0 for SMD. All statistical analyses were made assuming a two-sided test at the 95 % level of significance using Medcalc software (Microsoft, USA).

## Results

Search syntax described previously was used and total of 2,807 results were obtained (2,793 from PubMed and 14 from Cochrane library). Duplicate articles (n=6) were removed and 2,801 articles were reviewed. A total of 2,758 articles were excluded based on title and abstract review. Forty three articles were reviewed on the basis of their full text. According to exclusion criteria, 29 articles were rejected. Finally, 14 articles were included in this meta-analysis with total of 732 patients [15, 21], [22], [23], [24], [25], [26], [27], [28], [29], [30], [31], [32], [33] [Table 1]. Preferred Reporting Items for Systematic Review and Meta-Analysis (PRISMA) flowchart and selection criteria is shown in Figure 1. The assessment of the quality of studies in this meta-analysis was done by Newcastle-Ottawa Quality Assessment Scale and all studies showed a score of five stars and above indicating ‘good studies’.

**Table 1:** Overview of studies included in the meta-analysis.

Sr. No.	Author	Country	Year	Study period	Study duration	Type of MAS	HIPEC/EPIC	Total	M-CRS	O-CRS	Type of study	Comparability of both groups	HIPEC/EPIC protocols
1	Sheng-Chi Chang	Taiwan	2022	April 2016–April 2021	60 months	Lap and HAL	HIPEC	68	34	34	Retrospective	Propensity matched	[1] Oxa 460 mg/m^2^ (42) 60 min
													[2] MMC 35 mg/m^2^ (42) 90 min
													[3] MMC 3.3 mg/m^2^/L+Cis 25 mg/m^2^/L (42) 60 min
2	Chae Yun Cho	USA	2022	Jan 2014 to Aug 2020	80 months	Lap	HIPEC	191	31	160	Retrospective	Matched	[1] MMC 10 mg/L+Cis 50 mg/m^2^
													[2] Cis 50 mg/m^2^+Doxo 15 mg/m^2^ – MM
													[3] Cis 100 mg/m^2^-ovary
3	Chong Wang	China	2022	Jan 2018 to Dec 2021	47 months	Lap	HIPEC	66	33	33	Retrospective	Propensity matched	Cisplatin 60–80 mg/m^2^ (41) 60 min
4	L. Rodriguez-Ortiz	Spain	2020	Jan 2009 to July 2019	126 months	Lap	HIPEC	60	18	42	Retrospective	Matched	[1] MMC 30 mg/m^2^ (42) 60 min-intestinal
													[2] Pacli 120 mg/m^2^ (42) 60 min-ovarian
													[3] Cis100 mg/m^2^+Doxo 30 mg/m^2^ (42) 60 min-MM
5	Haytham Abudeeb	UK	2019	Jan 2003 to Jan 2018	180 months	Lap	HIPEC	84	55	29	Retrospective	Matched	MMC 35 mg/m^2^ (42) 90 min
6	Shruti Koti	US	2019	July 2014 to Feb 2019	55 months	Robotic+Lap	HIPEC	33	9	24	Retrospective	Matched	MMC 40 mg (42) 90 min
7	Frederic Mercier	France	2018	Mar 2009 to June 2017	75 months	Lap	HIPEC	43	32	11	Retrospective	Matched	[1] Cis 50 mg/m^2^+Doxo15 mg/m^2^ 90 min-MM
													[2] Cis 50 mg/m^2^+MMC 35 mg/m^2^ 90 min-PMP
													[3] Oxa 360 mg/m^2^ 30 min-PMP
8	Sang Hun Ha	Korea	2018	Nov 2004 to Dec 2017	157 months	Lap	HIPEC/EPIC	63	42	21	Retrospective	Comparable	[1] EPIC D1 MMC 10 mg/m^2^/day D2–D5 5-Fluorouracil 700 mg/m^2^/day
													[2] HIPEC MMC 35 mg/m^2^ (41–43) 90 min
9	George I. Salti	USA	2017	Mar 2015 to Aug 2017	29 months	Lap + HAL	HIPEC	22	11	11	Retrospective	Matched	NA
10	A. Fagotti	Italy	2014	May 2005 to June 2013	97 months	Lap + robotic	HIPEC	22	11	11	Retrospective	Matched	[1] Oxa 460 mg/m^2^ (41.5) 30 min
													[2] Cis75 mg/m^2^ (41.5) 60 min
11	Rebecca Fish	UK	2013	Dec 2010 to Dec 2012	24 months	Lap and HAL	HIPEC	17	10	7	Retrospective	Matched	MMC 35 mg/m^2^ (42) 90 min
12	G. Passot	France	2013	Jan 2011 to Nov 2012	22 months	Lap	HIPEC	16	8	8	Retrospective	Matched	[1] Oxa 360 mg/m^2^ (42) 30 min-PMP
													[2] MMC35 mg/m^2^+cis45 mg/m^2^ (42) 90 min-PMP
													[3] Cis20 mg/m^2^+Doxo15 mg/m^2^ (42) 60 min-MM
13	Soo Yeun Park	Korea	2013	Nov 2004 to Feb 2010	63 months	Lap	EPIC	29	15	14	Retrospective	Matched	EPIC D1 MMC 10 mg/m^2^/day … D2–D5 5-Fluorouracil 700 mg/m^2^/day
14	Jesus Esquivel	USA	2011	Oct 2008 to Jan 2010	15 months	Lap	HIPEC	18	10	8	Retrospective	Comparable	[1] Cis+Doxo (43) 90 min
													[2] MMC(43) 90 min

There was a total 732 patients of which 319 (43.57 %) were in the study group i.e. minimal access CRS and HIPEC/EPIC and 413 (56.43 %) were in the control group i.e. open CRS and HIPEC/EPIC group. The median age was 55.7 (48.1–63.3) years in the M-CRS group and 53.3 (47.3–59.3) years in the O-CRS group. The female to male ratio was 1.31:1 and 1.67:1 whereas median BMI was 26.4 and 27.5 in the M-CRS and O-CRS groups, respectively. As only studies with matched proportion of population were considered, there was little heterogeneity in the demography. Other demographic and patient factors are given in the table (Table 2). Of all the 732 patients 48.9 % had appendix as primary, 35.1 % had colorectal primary, 5.9 % had ovarian primary and 5.5 % had mesothelioma as primary. Remaining patients had miscellaneous primaries. Majority of patients in the study had PCI <10. Disease characteristics of both groups are given in (Table 3).

**Table 2:** Demographic characteristics of patients in the studies.

Sr. No.	Author	Age (M)	Age (O)	Female (M)	Female (O)	BMI (M)	BMI (O)	Prior sx (M)	Prior sx (O)	Neoadj (M)	Neoadj (O)
1	Sheng-Chi Chang	55 (33–81)	53 (38–75)	19 (55.8)	14 (41.1)	21.9	24.2	23 (67.6)	25 (73.5)	13 (38.2)	17 (50)
2	Chae Yun Cho	57 (29–78)	55 (26–86)	16 (52)	108 (68)	25.7	25	NA	NA	9 (29)	106 (66)
3	Rebecca Fish	56 (30–75)	53 (35–62)	6 (60)	3 (42.8)	30 (24–38)	30 (20–38)	10 (100)	7 (100)	0	0
4	Haytham Abudeeb	55 (44–64)	50 (43–62)	32 (58)	14 (48)	NA	NA	55 (100)	29 (100)	0	0
5	Shruti Koti	57.34	53.3	8 (88.9)	19 (79.2)	27.03	26.19	NA	NA	1 (11.1)	3 (12.5)
6	Jesus Esquivel	55 (45–65)	54.4	7 (70)	7 (87.5)	28 (21–35)	27.5	10 (100)	8 (100)	10 (100)	1 (12.5)
7	George I. Salti	58.5	58.5	4 (36.4)	3 (27.3)	26.4	29.02	NA	NA	3 (27.3)	3 (27.3)
8	Frederic Mercier	44.9	54.9	20 (62.5)	8 (72.7)	21.2	23.9	NA	NA	NA	0
9	L. Rodriguez-Ortiz	56 (51–61)	61.5	NA	NA	26.11	27.7	11 (61.1)	24 (57.14)	8 (44.4)	30 (71.42)
10	G. Passot	47.5 (26–71)	56 (43–70)	6 (75)	4 (50)	NA	NA	8 (100)	8 (100)	NA	NA
11	Chong Wang	53 (47–63)	53 (47–63)	17 (51.5)	14 (42.4)	NA	NA	19 (57.6)	23 (69.7)	NA	NA
12	A. Fagotti	57 (45–68)	51 (36–60)	11 (100)	11 (100)	24 (19–28)	26 (21–29)	11 (100)	11 (100)	11 (100)	11 (100)
13	Soo Yeun Park	52 (21–70)	53 (24–68)	9 (60)	12 (85.7)	22 (19–30)	22 (15–28)	NA	NA	0	0
14	Sang Hun Ha	55.7	51.2	16 (38.1)	15 (71.4)	23.7	22.3	8 (19)	2 (9.5)	1 (2.4)	2 (9.5)

**Table 3:** Disease characteristics and histology.

Sr. No.	Author	PCI<10 (M)	PCI<10 (O)	App (M)	App (O)	CRC (M)	CRC (O)	MM-(M)	MM-(O)	Ovary (M)	Ovary (O)	Other (M)	Other (O)
1	Sheng-Chi Chang	79.4	27 (79.4)	AdenoCa	AdenoCa	AdenoCa	AdenoCa	0	0	0	0	0	0
				3 (8.8)	2 (5.9)	31 (91)	32 (94)						
2	Chae Yun Cho	31 (100)	160 (100)	LAMN 13 (42)	LAMN 41 (26 %)	AdenoCA	AdenoCA	0	Epitheliod MM	0	High grade Serous	AdenoCa	AdenoCa
				AdenoCa 11 (35.4)	AdenoCA 27 (16.8)	5 (16)	58 (36)		13 (8)		11 (7)	2 (8)	10 (6)
3	Rebecca Fish	10 (100)	7 (100)	LAMN	LAMN	0	0	0	0	0	0	0	0
				17 (100)	7 (100)								
4	Haytham Abudeeb	55 (100)	29 (100)	LAMN	LAMN	0	0	0	0	0	0	0	0
				55 (100)	29 (100)								
5	Shruti Koti	9 (100)	24 (100)	LAMN AdenoCa	LAMN AdenoCa	AdenoCa	AdenoCa	0	0	0	0	Gastric AdenoCa1 (11.1)	GastricAdenoCa 3 (12.5)
				5 (55.6)	9 (37.5)	2 (33.3)	11 (46)						SB AdenoCa 1 (4.2)
6	Jesus Esquivel	10 (100)	8 (100)	LAMN	LAMN	0	AdenoCa	EpitheloidMM	EpitheloidMM	0	0	1	Primary peritoneal carcinoma
				8 (80)	5 (62.5)		1 (12.5)	1 (10)	1 (12.5)				1 (12.5)
7	George I. Salti	11 (100)	11 (100)	LAMN	LAMN	AdenoCa	AdenoCa	0	0	0	0	0	0
				6 (54.6)	4 (36.4)								
				AdenoCa	AdenoCa	3 (27.3)	3 (27.3)						
				2 (18.2)	4 (36.4)								
8	Frederic Mercier	32 (100)	10 (91)	LAMN	LAMN	0	0	Multicystic mesothelioma	Multicystic mesothelioma	0	Mucinous	0	0
				20 (65.2)	6 (54.54)			12 (37.5)	3 (27.3)		2 (18.2)		
9	L. Rodriguez-Ortiz	18 (100)	42 (100)	LAMN	LAMN	AdenoCa	AdenoCa	EpitheloidMM	EpitheloidMM	High grade serous	High grade serous	Endometrial	Endometrial 4 (9.8)
				2 (11.8)	6 (14.6)	6 (35.3)	13 (32)	2 (11.8)	2 (4.9)	6 (35.3)	15 (36.6)	1 (5.9)	Signet ring cell 1 (2.4)
10	G. Passot	8 (100)	6 (75)	LAMN	LAMN	0	0	Multicystic mesothelioma	Multicystic mesothelioma	0	0	0	0
				5 (62.5)	5 (62.5)			3 (37.5)	3 (37.5)				
11	Chong Wang	33 (100)	33 (100)	LAMN	LAMN	0	0	0	0	0	0	0	0
				33 (100)	33 (100)								
12	A. Fagotti	11 (100)	11 (100)	0	0	0	0	0	0	High grade serous	High grade serous	0	0
										11 (100)	11 (100)		
13	Soo Yeun Park	NA	NA	0	0	MD AdenoCa	MD AdenoCa	0	0	0	0	0	0
						11 (73.3)	12 (85.7)						
						PD AdenoCa	PD AdenoCa						
						4 (26.7)	2 (26.7)						
14	Sang Hun Ha	42 (100)	21 (100)	0	0	WD/MD AdenoCa	WD/MD AdenoCa	0	0	0	0	0	0
						35 (83.3)	16 (76.2)						
						PD/mucinous/signet AdenoCa	PD/mucinous/signet AdenoCa						
						7 (16.7)	5 (23.8)						

**Figure 2:**
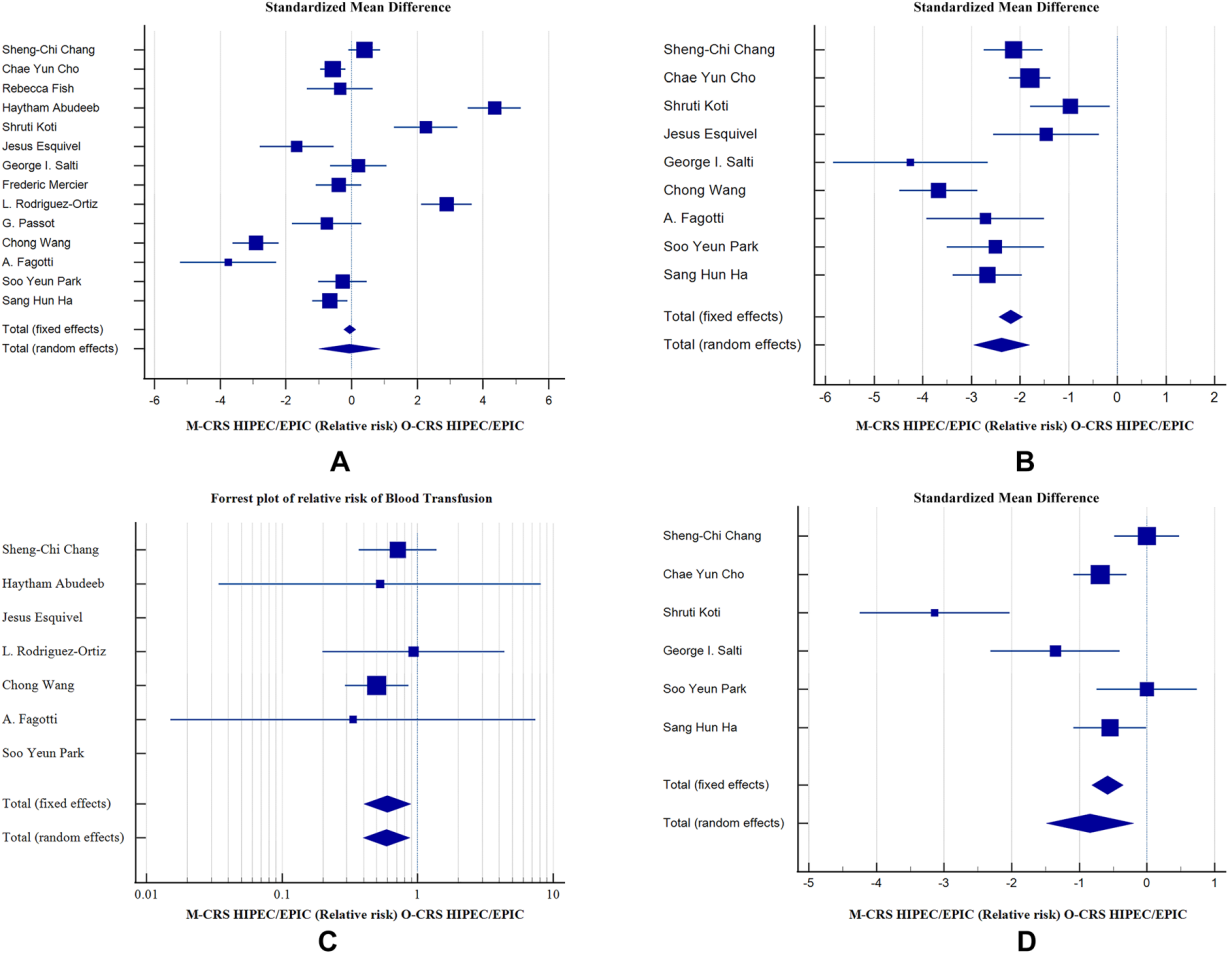
Forrest plots of relative risk and standardized mean difference of different peri-operative outcomes between M-CRS and O-CRS group. (A) Duration of surgery, (B) estimated blood loss, (C) blood transfusions; (D) bowel recovery.

The parameter of duration of surgery from incision to closure was reported by all 14 studies. The statistical analysis for duration of surgery in minutes between the M-CRS and O-CRS groups showed significant heterogeneity by the Higgins and Thompsons method with I^2^ 95.99 % (95 % CI for I^2^ 94.54 to 97.05, p<0.0001) hence random effects model of DerSimonian and Laird was used to calculate relative risk. Funnel plot and Egger’s regression test were used to test publication bias which was 0.7372 (p=0.8672, 95 % CI −8.668 to 10.143). The Standardized Mean Difference for duration of surgery was −0.0643 (95 % CI −0.993 to 0.865, p=0.892). No significant difference was seen between two groups (Figure 2A).

Median estimated blood loss was reported by nine studies. The Standardized Mean Difference (SDM) for estimated blood loss in mL between M-CRS and O-CRS was SMD=−2.379, p<0.001 (95 % CI −2.952 to −1.805) with random effects model (I^2^ 79.30 %, 95 % CI 61.22 to 88.95, p<0.0001). Publication bias was −2.6251 (95 % CI −7.4531 to 2.2029 with p=0.2109). There was significantly less blood loss in the M-CRS group (Figure 2B).

Number of patients who required blood transfusions were reported in seven studies out of 14. The relative risk for blood transfusion in M-CRS and O-CRS was 0.598 with significant p-value of p=0.011 (95 % CI 0.402 to 0.889) fixed effects model with I^2^ 0.00 % (95 % CI 0.00 to 32.90, p=0.8835). Publication bias 0.07921 (95 % CI −1.5120 to 1.6704, p=0.8842). There were significantly less number of blood transfusions in the M-CRS group as compared to O-CRS group (Figure 2C).

Parameter of bowel recovery in terms of number of days to pass flatus or number of days to start oral feeding were reported by six studies. The standardized mean difference for bowel recovery was SMD=−0.843, p=0.01 (95 % CI −1.487 to −0.2) with I^2^ 85.24 % (95 % CI 69.76 to 92.79, p<0.0001) and publication bias −4.3775 (95 % CI −12.5461 to 3.7910, p=0.2110). There was significantly better bowel recovery in the M-CRS group (Figure 2D).

**Figure 3:**
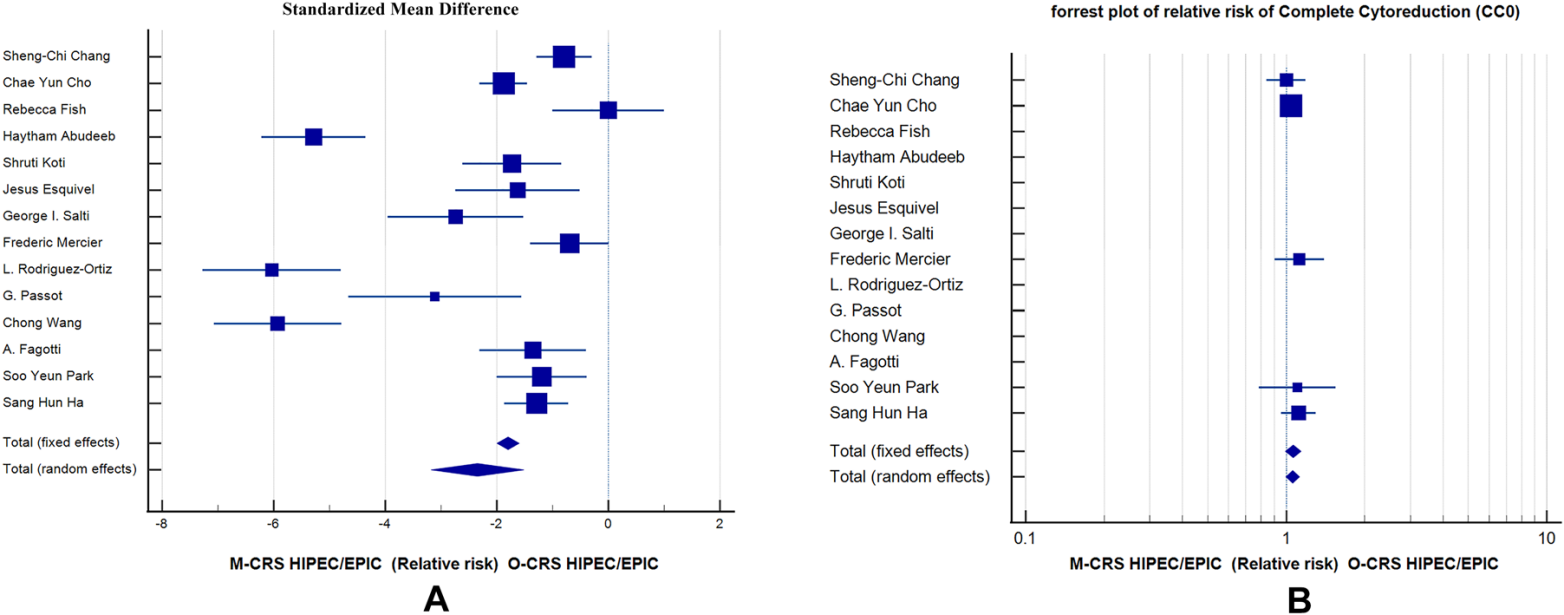
(A) Forrest plot of SMD of hospital stay in days in M-CRS and O-CRS groups, (B) Forrest plot of relative risk of completeness of cytoreduction score 0 in M-CRS and O-CRS groups.

Number of days in hospital post-surgery was reported in all studies. The Standardized Mean Difference was SMD=−2.348, p<0.001 (95 % CI −3.178 to −1.519) by random effects model. I^2^ 93.78 % (95 % CI 91.15 to 95.63, p<0.0001) and publication bias −5.6364 (95 % CI −11.6817 to 0.4089 p=0.0650). Significantly less number of days in hospital was spent in the M-CRS group (Figure 3A).

**Figure 4:**
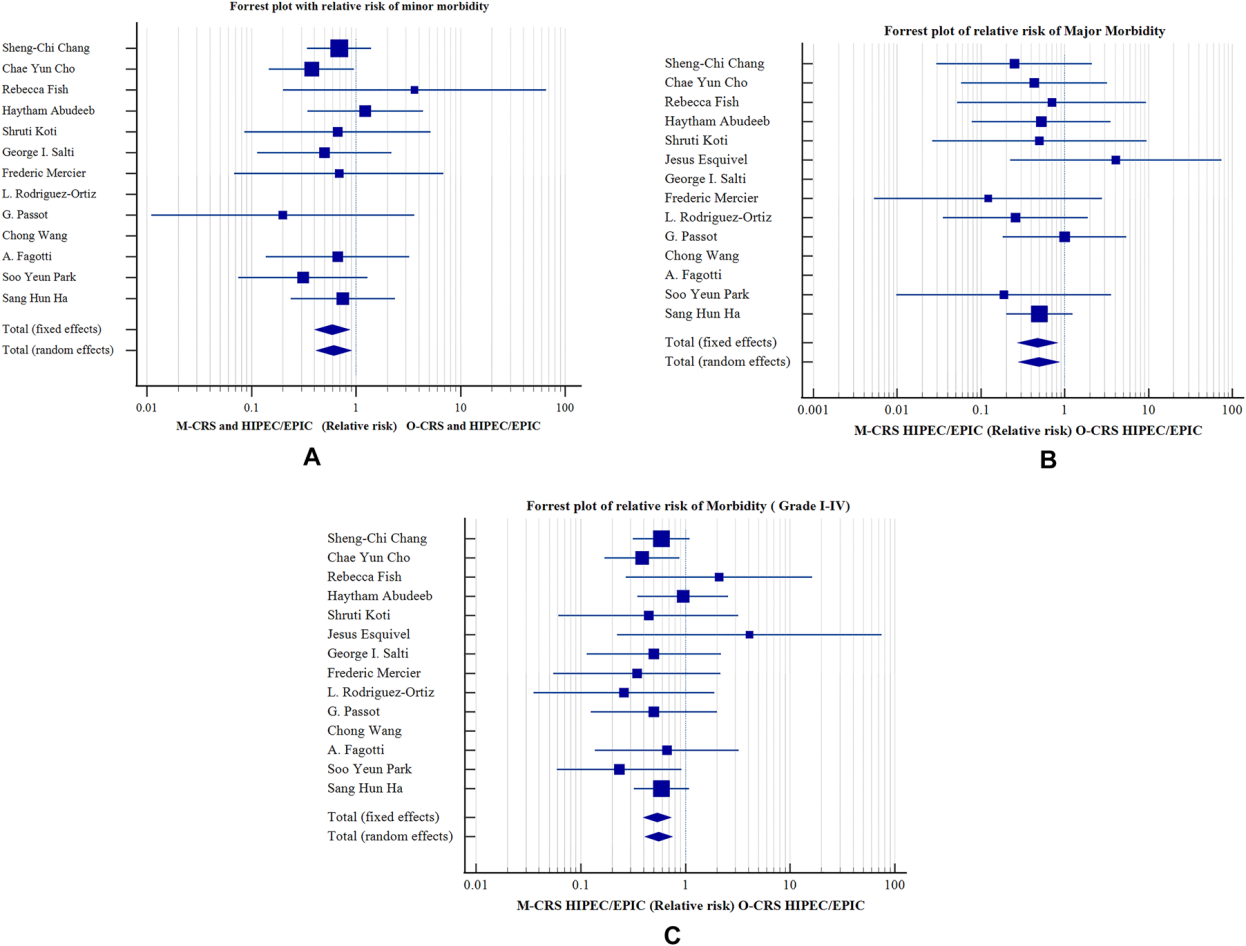
Forrest plots of relative risk of morbidity between M-CRS and O-CRS groups. (A) Minor morbidity, (B) major morbidity, (C) total morbidity.

Morbidity of procedures was reported in 13 studies. Grade I and Grade II Clavien–Dindo morbidities were considered minor morbidity and Grade III and Grade IV Clavien–Dindo morbidities were considered major morbidity. The relative risk of minor morbidity was 0.592 (95 % CI 0.401 to 0.872 with p=0.008) with fixed effects model with I^2^ 0.00 % (95 % CI 0.00 to 27.34, p=0.8589) and publication bias 0.1479 (95 % CI −1.1630 to 1.4589, p=0.8043). The relative risk for major morbidity was 0.474 (95 % CI 0.273 to 0.821 with p=0.08) with fixed effects model with I^2^ 0.00 % (95 % CI 0.00 to 17.17 with p=0.9052) and publication bias −0.1432 (95 % CI −1.3937 to 1.1073, p=0.8014). The relative risk of total morbidity (Grade I to IV) was 0.538 (95 % CI 0.395 to 0.731 with p<0.001) with I^2^ 0.00 % (95 % CI 0.00 to 34.48, p=0.7905) and publication bias 0.1626 (95 % CI −0.9860 to 1.3113, p=0.7611). This shows significantly less minor morbidity, major morbidity and total morbidity in M-CRS group as compared to O-CRS group (Figure 4A–C).

The parameter of completeness of cytoreduction score [CC score] were reported directly or indirectly in all 14 studies. Both groups had low peritoneal burden with mean PCI of <10. The relative risk for CC0 score for M-CRS vs. O-CRS groups was 1.064 (95 % CI 0.992 to 1.140 with p=0.083) with I^2^ 0.00 % (95 % CI 0.00 to 40.92, p=0.8570) and publication bias 0.4821 (95 % CI −1.3639 to 2.3281, p=0.4668). No significant difference is seen between M-CRS and O-CRS groups hence strengthening the feasibility and oncological equivalence of both methods (Figure 3B).

The oncological and survival outcomes are shown in the table (Table 4). All studies didn’t have long follow up hence the interpretation of the outcomes like disease free survival and overall survival is limited. Sheng-Chi Chang et al. in their study report 4 (11.8 %) early peritoneal recurrence (<12 months) in the M-CRS group [median follow-up 24.8 (1.1–56.3) months] and 10 (29.4 %) in the O-CRS group [median follow up 47 (4.9–61.3)]. He reports 3 years overall survival of 78.5 % and 63.8 % in the M-CRS and O-CRS groups respectively [21]. Frederic Mercier with median follow up of 33 (19.3–41.6) months in M-CRS and 19.9 (1.05–35) months in O-CRS reports 1 year and 5 year DFS of 100 % and 91.04 % in M-CRS and 100 % and 62.5 % in O-CRS groups respectively. He also reports 5-year survival of 100 % in both M-CRS and O-CRS groups [27]. L. Rodriguez-Ortiz in their study report DFS and OS similar in both groups but Park SY et al. report better DFS in M-CRS group with similar OS in both groups [28, 32]. Chong Wang et al. mentions that there was no difference in DFS in both groups, OS was not reached due to limited follow up. Sang Hun Ha reports 3-year peritoneal recurrence free survival, DFS and OS of 51 %, 39 %, 67  and 53 %, 33 % and 66 % in M-CRS and O-CRS groups [30, 33]. 30 day mortality in the M-CRS group was 0 % in 13 of 14 studies, only in one study by Ha et al. there was 90-day mortality of one patient (2.4 %) [33]. Thirty-day mortality in the O-CRS group was 1(7.1 %) by Park et al., 4(16.6 %) by Koti et al. and 90-day mortality was 1(4.8 %) in the study by Ha et al. [25, 32, 33] Overall, the survival outcomes were not evaluated statistically but limited number of studies that evaluated survival outcomes showed that they were similar.

**Table 4:** Survival outcomes of studies in the meta-analysis.

Sr. No.	Author	Morbidity (M)	Morbidity (O)	Mortality (M)	Mortality (O)	Follow up (months) (M)	Follow up (months) (O)	DFS (M)	DFS (O)	OS (M)	OS (O)
1	Sheng-Chi Chang	10 (29.4)	17 (50)	0	0	24.8 (1.1–56.3)	47 (4.9–61.3)	No diff	No diff	3 years – 78.5	3 years – 63.8
2	Chae Yun Cho	5 (16)	67 (42)	0	0	38.4	38.4	No diff	No diff	No diff	No diff
3	Rebecca Fish	3 (30)	1	0	0	3.5 (0.2–12.9)	11.2 (0.2–21.5)	NA	NA	NA	NA
4	Haytham Abudeeb	9 (16.4)	5 (17.2)	0	0	27.6	27.6	NA	NA	NA	NA
5	Shruti Koti	1 (11.1)	6 (25)	0	4 (16.6)	19	19	NA	NA	NA	NA
6	Jesus Esquivel	2 (20)	0	0	0	8 (1–15)	8 (1–15)	NA	NA	NA	NA
7	George I. Salti	2 (18.2)	4 (36.36)	0	0	15.2 (3.5–26.9)	15.2 (3.5–26.9)	NA	NA	NA	NA
8	Frederic Mercier	2 (6.3)	2 (18.2)	0	0	33 (19.3–41.6)	19.9 (1.05–35)	1 year – 100, 5 years – 91.04	1 year – 100, 5 years – 62.5	1 year 100, 5 years – 100	1 year 100, 5 years 100
9	L. Rodriguez-Ortiz	1 (5.6)	9 (21.4)	0	0	24	95	15.5 (8.75–23) months, 2 years DFS – 71.4 %	33.5 (12–94.5) months, 2 years DFS – 63.7 %	2 years OS – 100 %	2 years OS – 97.3 %
10	G. Passot	2 (25)	4 (50)	0	0	6.4 (1.4–21.26)	6.4 (1.43–21.26)	Not reached	Not reached	Not reached	Not reached
11	Chong Wang	0	0	0	0	20 (1–37)	20 (1–37)	No diff	No diff	Not reached	Not reached
12	A. Fagotti	2 (18.2)	3 (27.3)	0	0	NA	NA	NA	NA	NA	NA
13	Soo Yeun Park	2 (13.3)	8 (57.1)	0	1 (7.1)	31 (9–69)	20.5 (2–46)	3 years – 42.9, 3 years(CC0) – 53.8	3 years – 23.1, 3 years(CC0) – 27.3	3 years – 43.5, 3 years(CC0) – 58.6	3 years – 50.8, 3 years(CC0) – 56.1
14	Sang Hun Ha	13 (30.9)	11 (52.3)	90 day – 1 (2.4)	90 day – 1 (4.8)	33.4	19.7	3 years – 39 %	3 years – 33 %	3 years – 67, median OS – 57 months	3 years – 66 %, median OS: 38 months

## Discussion

Peritoneal surface malignancies comprise of a heterogenous group of malignancies which can be primary or secondarily spread to peritoneum from other organs with unique proclivity of peritoneal dissemination [34]. Cytoreductive surgery and intra-peritoneal chemotherapy in the form of HIPEC/EPIC/PIPAC has been proven to improve overall survival in these patients [35]. Even in rare primary malignancies like sarcomas, GIST, etc. with peritoneal only dissemination, CRS and HIPEC has shown benefit [36]. At present, open CRS and HIPEC has major morbidity of 22–34 % and mortality rates of 0.8–4.1 % [37]. With the standardisation of this procedure, it becomes necessary to reduce the surgery related morbidity with the use of modern technologies without affecting the oncological outcomes. Minimal access surgery has revolutionised this concept in various oncological fields by significantly decreasing morbidity and mortality. Earlier there were concerns regarding increased peritoneal dissemination of cancer cells by laparoscopy but prospective randomised trials have shown no difference in port site or peritoneal recurrences [38].

The minimal access approach has a theoretical advantage of decreased adhesion formation post-surgery which may show benefit according to ‘Fibrin entrapment hypothesis’ [39]. Also decreased hospital stay and complications in the post-operative period leads to decreased over all costs for the patient and hospital [25, 40, 41]. Another important advantage of minimal access surgery is decreased time to adjuvant chemotherapy due to overall early recovery of patients and has shown to increase disease free survival [21]. This has a lesser role in low grade malignancies like appendiceal mucinous neoplasms and multi cystic mesothelioma but has a major impact on patients with high grade primaries like ovary and colorectal malignancies [42, 43].

Initially the use of laparoscopy was limited to calculating PCI, to evaluate candidates for complete cytoreduction and therapeutic drainage of mucinous ascites for palliation and symptom control [44, 45]. In 2005, Ferron et al. first described feasibility of laparoscopic CRS and HIPEC in animals [46]. In 2011, Esquivel et al. were first to show feasibility of laparoscopic CRS and HIPEC in patients with peritoneal surface malignancies [16]. Since then many case series and small retrospective studies have shown feasibility of minimal access cytoreductive surgery and HIPEC. Further in this regard, Koti et al. and Fagotti et al. have shown feasibility of robotic CRS and HIPEC in their studies [25, 31].

To our knowledge this meta-analysis is first of its kind which directly compares outcomes of minimal access vs. open CRS and HIPEC/EPIC across different peritoneal surface malignancies. This meta-analysis includes studies with laparoscopic, hand assisted laparoscopic and robotic CRS and HIPEC/EPIC procedures. The PSOGI international collaborative registry and ASPSM multi-institution analysis describe outcomes of patients undergoing laparoscopic CRS and HIPEC in various peritoneal surface malignancies but lack direct comparison with open CRS procedures [17, 18].

In this meta-analysis peri-operative outcomes were compared in terms of duration of surgery, estimated blood loss, number blood transfusions, days for bowel recovery (time to pass flatus/time to start orals), hospital stay and morbidity [(minor=Grade I and Grade II) (major=Grade III and Grade IV) and (total=Grade I–IV)] according to Clavien–Dindo classification [47]. Technical feasibility outcomes were compared in terms of completeness of cytoreduction score 0. Survival outcomes were not evaluated statistically.

A Arjona-Sanchez et al. in the PSOGI international collaborative registry have described outcomes of 143 patients with laparoscopic CRS and HIPEC and have shown similar peri-operative outcomes to our meta-analysis of 319 patients of M-CRS group [17]. Our meta-analysis shows that peri-operative outcomes in terms of blood loss, blood transfusion, bowel recovery, hospital stay and morbidity are significantly better in the M-CRS group while duration of surgery have no significant difference. These findings are similar to the peri-operative outcomes of COLOR II trial and COREAN trial for colorectal cancers and MISSION trial for ovarian cancer comparing minimal access surgery to open surgery [48], [49], [50]. The oncological safety of procedure in terms of CC0 had no significant difference in both groups when the median PCI was low i.e. <10. This infers that minimal access approach can be used in patients with low PCI.

As many of the studies in this meta-analysis were retrospective cohort studies, the follow up period was less and survival outcomes like disease free survival, peritoneal recurrence free survival and overall survival could not be compared across both groups with accuracy. But, few studies like Chang et al., Cho CY et al., Mercer et al., Rodriguez-Ortiz et al., Wang et al. and Ha et al. have shown that disease free survival and overall survival in both groups were similar [21, 22, 27, 28, 30, 33]. Interestingly, Chang et al. have shown less early peritoneal recurrence (defined as peritoneal recurrence in less than 12 months) in the M-CRS group than O-CRS group (11.8 vs. 29.4 %) [21]. Similar results are shown by Salt et al. and Rodríguez-Ortiz et al. [26, 28]. Also, Park et al. have shown better 3-year DFS in the M-CRS group than the O-CRS group (53.8 vs. 27.3 %) in patients in whom complete cytoreduction was achieved (CC0) [32]. These finding may be attributed to the higher pressures reached during the minimal invasive HIPEC procedure which has shown to increase penetration and cytotoxicity of chemotherapeutic agents [51], [52], [53].

Finally, we believe that the strength of our meta-analysis is that a fair number of patients (732 patients, M-CRS 319 and O-CRS 413) were included in the study and comparative analysis was made. Also, the demographic characteristics of the patients in both groups were comparable since only studies with matched population were included. The major limitation of this meta-analysis is that, majority of studies barring a few are retrospective cohort studies. This is due to paucity of comparative work, relatively novel approach of minimal access in cytoreductive surgeries and lack of randomised controlled trials. Another limitation is that survival outcomes were not calculated statistically as only few studies mentioned survival outcomes and different studies had different primaries and histology.

## Conclusions

This meta-analysis shows that minimal access cytoreductive surgery and HIPEC/EPIC is feasible and has better peri-operative outcomes compared to open procedure in patients having limited PCI peritoneal surface malignancies. However, further collaborative, prospective studies and randomised trials are warranted to confirm the results and for better assessment of survival outcomes.
